# Mechanical rheological model on the assessment of elasticity and viscosity in tissue inflammation: A systematic review

**DOI:** 10.1371/journal.pone.0307113

**Published:** 2024-07-15

**Authors:** Jotham Josephat Kimondo, Ramadhan Rashid Said, Jun Wu, Chao Tian, Zhe Wu

**Affiliations:** 1 School of life Science and Technology, University of Electronic Science and Technology of China, Chengdu, China; 2 School of Medical Imaging, North Sichuan Medical College, Nanchong, Sichuan, China; 3 Department of Women’s Health, Sichuan Cancer Hospital, Chengdu, China; 4 Tianfu Jincheng Laboratory, City of Future Medicine, Chengdu, China; Gdańsk University of Technology: Politechnika Gdanska, POLAND

## Abstract

Understanding the extent of inflammation is crucial for early disease detection, monitoring disease progression, and evaluating treatment responses. Over the past decade, researchers have demonstrated the need to understand the extent of inflammation through qualitative or quantitative characterization of tissue viscoelasticity using different techniques. In this scientific review, an examination of research on the association between elasticity and Viscosity in diseases, particularly as tissue inflammation progresses, is conducted. A review of utilizing mechanical rheological models to characterize quantitative viscoelastic parameters of normal and inflamed tissues is also undertaken. Based on inclusion and exclusion criteria, we identified 14 full-text studies suitable for review out of 290 articles published from January 2000 to January 2024. We used PRISMA guidelines for the systematic review. In the review, three studies demonstrated the criterion used by the researchers in identifying the best rheological model. Eleven studies showed the clinical application of the rheological model in quantifying the viscoelastic properties of normal and pathological tissue. The review quantified viscoelastic parameters for normal and pathological tissue across various soft tissues. It evaluated the effectiveness of each viscoelastic property in distinguishing between normal and pathological tissue stiffness. Furthermore, the review outlined additional viscoelastic-related parameters for researchers to consider in future stiffness classification studies.

## 1. Introduction

Inflammation is associated with cell death, particularly necrosis, and is often observed in various pathological conditions and diseases [1]. Understanding the extent of inflammation is crucial for early disease detection, monitoring disease progression, and evaluating treatment responses. Inflammation, the body’s protective mechanism against injury or infection, involves the interaction of the solid phase (collagenous extracellular matrix and cells) and the fluid phase (containing interfibrillar fluid) in soft tissues, defining their biomechanical properties [2–4]. In response to tissue injury, characterized by necrosis and inflammation, the body initiates healing by releasing inflammatory mediators [4]. These mediators help to remove pathogens and damaged cells while promoting tissue repair. However, uncontrolled inflammation can contribute to diseases like benign and malignant tumors (cancer) [5–7]. Therefore, researchers utilize invasive and noninvasive methods to diagnose these inflammatory diseases. Pathologists deploy invasive procedures (biopsy) as the gold standard to detect and assess the inflammation level within soft tissues [8–10]. Pathologists have established a scoring system that helps to grade the inflammation level of the tissue. Examples of the scoring system exercised by pathologists include METAVIR [8], TNM, and Gleason [9] scores for liver, breast, and prostate, respectively. For decades, biopsy has been regarded as the gold standard for detecting inflammation but suffers a limitation of sampling error [8, 9]. The procedure involves taking a portion of an inflamed tissue and giving the result as an average of the whole soft tissue.

**Fig 1:**
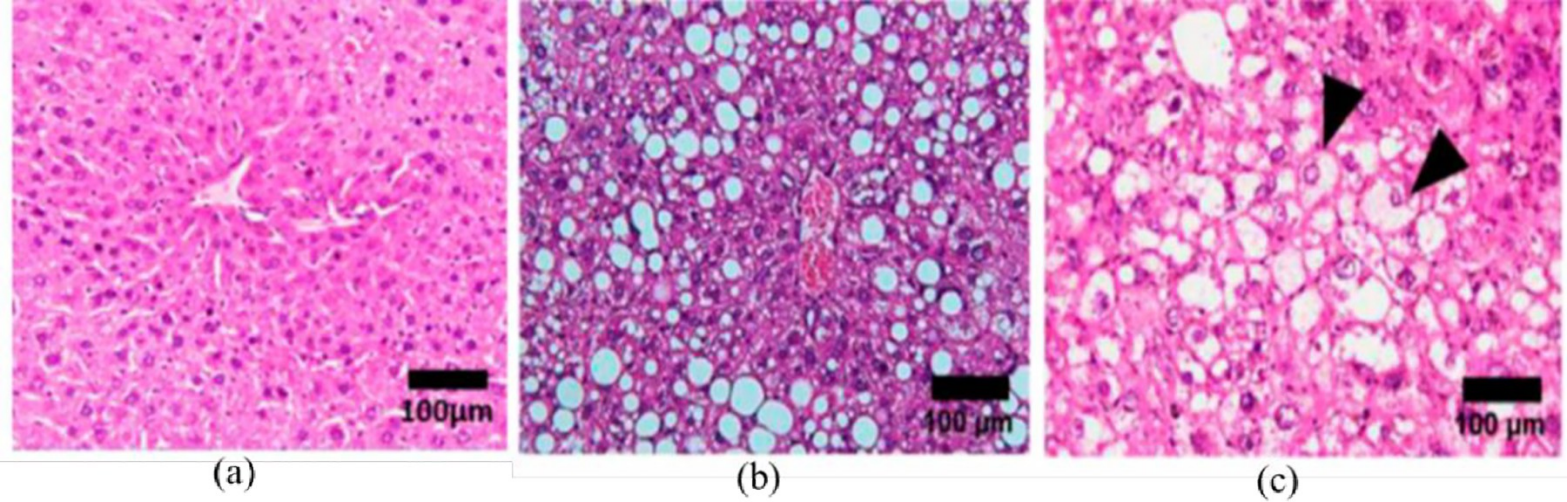
Micrographs of a Human liver changes due to inflammation (a) healthy tissue with well-arranged and densely packed Hepatocytes,(b) Hepatocytes with pathologies in the early stage of Non- alcoholic fat liver disease, (c) inflammatory cells and hepatocytes with altered architecture due to uncontrolled lipid accumulation, adapted from [17].

Moreover, the biopsy procedure carries risks such as postoperative bleeding, surrounding skin damage, and wound infection [11]. It is also hard to perform, especially for deeply located tissue like the prostate [12]. As a result, scientists have demonstrated noninvasive methods to quantify the biomechanical properties of soft tissue, which are directly correlated with the degree of inflammation. Elasticity and Viscosity are biomechanical properties vital in detecting diseases, monitoring disease progression, and noninvasively assessing treatment responses [3, 13]. Elastic tissue deforms swiftly under strain and returns to normal after removing the load [14]. In contrast, viscous tissue resists deformation in proportion to the loading rate [14]. Soft tissues, in particular, are regarded as viscoelastic materials as they contain both elastic and Viscosity properties [15, 16]. The research conducted by Cheng et al.[17] explains the changes in tissue structure and biomechanical properties caused by inflammation, as shown in Fig 1. Their findings indicate that uncontrolled inflammation can lead to significant structural changes within tissues, potentially causing tissue rupture [8, 17]. Such ruptures are linked to the development of various diseases, highlighting the importance of controlling inflammation to maintain tissue integrity and prevent disease. For the early stage of the disease, these biomechanical properties of soft tissue mirror the inflammatory response seen in inflamed tissue. Thus, the precise extraction of viscoelastic properties to distinguish between healthy, inflamed, and diseased tissues in the early stages remains uncertain for noninvasive techniques like ultrasound elastography [18].

### 1.1 Ultrasound shear wave elastography: An overview

Recently, Researchers have applied ultrasound shear wave elastography (USWE) to assess diseases in soft tissue like the liver, prostate, Thyroid, Bladder, kidney, and lung [17, 19–23]. USWE seeks to investigate the viscoelastic properties in a specific area of interest that influence the flow of shear waves [16]. This movement is related to changes in the viscoelastic characteristics of soft tissue induced by various diseases. Quantifying the dynamic behavior of soft tissue using USWE is a necessary technique to understand physiological and pathological conditions. Despite its successes, USWE faces challenges characterizing the early stages of soft tissue diseases, mainly when inflammation is a crucial factor [10, 24]. The challenge lies in the subtlety of inflammatory changes during the initial phases of pathology. In the progression of soft tissue diseases, inflammation plays a key role, and alterations in tissue viscosity and elasticity intricately connect to these early stages. The dynamic and heterogeneous nature of inflammation challenges elastography, as normal tissue variations might overshadow the nuanced changes [10]. Several studies have showcased the capability of USWE to characterize the advanced stage of the disease. For example, for patients with chronic viral hepatitis, USWE stands out as the primary choice for initially evaluating the extent of liver fibrosis in untreated patients and excluding the presence of advanced disease [19]. It’s crucial to note that while USWE is excellent for assessing the severity of fibrosis and identifying potential progressive conditions, it is unsuitable for actual fibrosis staging [19, 25]. The current method employing shear wave speed propagation to estimate soft tissue stiffness focuses solely on overall stiffness [15]. Point Shear Wave Elastography (p-SWE), also known as Quantitative Ultrasound Elastography (QUS), Acoustic Radiation Force Impulse Imaging, and 2D-Shear Wave Elastography (2D-SWE), works by sending a focused ultrasound pulse into the tissues and measuring the speed of resulting shear waves emanating from the focal zone [25]. The shear speed correlates with the stiffness of the medium, and Shear Wave Speed (SWS) rises as soft tissue becomes stiffer [26]. With Eq 1 below, a standard stiffness of the soft tissue is estimated using QUS.


$$E=3\rho v_{s}^{2}$$ (1)

Whereas E represents Young’s modulus that quantifies stiffness, *ρ* signifies tissue density (approximately assumed as 1g cm−3 for soft tissues), and Vs denotes shear wave speed. Despite the effectiveness of QUS in quantifying soft tissue stiffness, no recommended cutoff value for SWS distinguishes between normal and abnormal tissue [26].

The above methods have considered only the biological tissue’s elastic behavior to characterize stiffness. Ignoring the viscosity component in stiffness characterization might introduce errors in estimating the stiffness as biological tissues are viscoelastic mediums [27]. Viscoelastic medium is complex (having real and imaginary) and Frequency-dependent [28]. The viscous component introduces a dissipative (imaginary) behavior of biological tissue and is responsible for dispersion. Therefore, for a biological tissue, the equation below gives a complex Young’s modulus of the longitudinal wave within a tissue.


$$E^{*}(w)=E_{0}(w)+iw\eta$$ (2)

Where E*(w) is the complex Young’s modulus, E_0_(w) is the elastic coefficient of the medium (real part), and η is the Viscosity, which represents a dispersive (imaginary) part of the medium.

Also, the equation below describes shear wave speed within the biological tissue.


$$v_{S}=\sqrt{\begin{array}{c} \frac{G^{*}(w)}{\rho } \end{array}}$$ (3)

G*(w) represents the complex shear modulus of the medium. As both components (Elastic and Viscosity) describe how shear wave propagates through biological tissue, one can obtain the shear wave speed from the relation between complex shear modulus and complex young modulus E* (w) given by Poisson’s ratio ν in the Eq 4 below [22, 29].


$$G^{*}(w)=\frac{E^{*}(w)}{2(1+v)}$$ (4)

We consider the biological tissues as an incompressible medium because they contain fluids; Therefore, it depicts Poisson’s ratio approximated to 0.5 [15], of which Eq 4 become

$$G^{*}(w)=\frac{E^{*}(w)}{3}$$ (5)

Upon substituting Eqs 2–5, the dispersion characteristics of the biological tissues become.


$$v_{S}=\sqrt{\begin{array}{c} \frac{E_{0}(w)+iw\eta }{3\rho } \end{array}}$$ (6)

The imaginary part reflects the attenuation or damping of the wave during its travel through the medium, indicating the rate at which the wave amplitude decreases over time [30]. In the context of liver assessment, studies utilize Vibration-Controlled Transient Elastography (VCTE) [14] to measure liver stiffness (LSM) and have considered the attenuation caused by fat accumulation. VCTE employs an external vibrator to generate shear waves systematically. The velocity of these shear waves is then translated into stiffness using Eq 1.

Additionally, VCTE offers a controlled attenuation parameter associated with steatosis measurements (Loss Modulus). The VCTE technique has shown promising results in the grading of steatosis. FibroScan 502 touch (Echosens, Paris, France) system, which deploys the VCTE technique, has been considered a standard noninvasive technique for detecting fatty liver. The machine uses a controlled attenuation parameter (CAP), expressed in decibels per meter (dB/m), to measure ultrasound attenuation through the fat medium [31]. Despite its capability, factors such as inflammation, BMI, fibrosis, and cholestasis still influence the CAP value, potentially affecting the accuracy of steatosis assessment. Moreover, it could not differentiate adjacent steatosis stages [14, 32].

One Limitation of USWE is that it ignores shear wave dispersion and instead uses a single group velocity measured over the shear wave bandwidth to estimate tissue elasticity [25, 32]. The center frequency and bandwidth of shear waves induced by acoustic radiation force depend on factors such as the ultrasound push beam (push duration, F-number) and the viscoelasticity of the medium [14]. As a result, different vendor ultrasound scanners may give different tissue elasticity measurements within the same patient. Studies have proposed various methods to evaluate shear wave dispersion to estimate tissue viscoelasticity better. One such method is the use of a rheological model, such as the Kelvin-Voigt model, Maxwell, standard linear solid, Springport, and fractional Voigt models, which is fitted to the shear wave dispersion to solve for the elasticity and Viscosity of tissue [29].

A rheological model consists of spring and dashpot presented by the equation that describes the flow (Viscosity) and deformation (Elasticity) behavior of materials under the influence of applied forces or stresses [28]. Therefore, as it consists of both elastic (Spring) and viscosity(dashpot) properties, rheological models are adopted in clinical applications to quantify the biomechanical properties of biological tissue. Due to their complex internal structure and different temporal scales, soft tissues often exhibit power-law viscoelasticity [33]. Rheological models are described based on the relaxation process [34]. Classical linear viscoelastic models, such as Kelvin Voigt (KV), Maxwell, Solid linear (SL), and Standard linear solid (Zener), are based on single relaxation. Fractional derivative models such as Kelvin Voigt fractional derivative (KVFD), Fractional -Zener, and Maxwell-FD capture multiple relaxation times or processes within tissue and reproduce power-low behavior seen in most tissues. Viscoelastic materials manifest themselves by their time and frequency-dependent characteristics. Therefore, the rheological model has also demonstrated their constitutive equation in frequency and time domains, as presented in Table 1.

**Table 1:** The constitutive equations of various rheological models describe the relationship between stress and strain in both the time and frequency domains. Each model has distinct parameters that quantify specific material properties.

Model	Parameters	Constitutive time domain equation	Constitutive Frequency domain equation
KV	E, *η*	$\sigma (t)=E\;\varepsilon (t)+\eta \left(\frac{d\varepsilon (t)}{dt}\right)$	$E^{*}(w)=E+\eta (i\omega )$
KVFD	E, *η*, *α*	$\sigma (t)=E\;\varepsilon (t)+\eta (\frac{d^{\alpha }\varepsilon (t)}{dt^{\alpha }})$	$E^{*}(w)=E+\eta {\left(i\omega \right)}^{\alpha }$
Maxwell	E, *η*	$\sigma (t)+(\frac{\eta }{E})\frac{d\sigma (t)}{dt}=\eta \frac{d\varepsilon (t)}{dt}$	$E^{*}(w)=\frac{(i\omega )\eta }{1+(i\omega )\frac{\eta }{E_{2}}}$
FD-Maxwell	E, *η*, *α*	$\sigma (t)+\eta \frac{d^{\alpha }\sigma (t)}{dt^{\alpha }}=E\;\varepsilon (t)$	$E^{*}(w)=\frac{{\left(i\omega \right)}^{\alpha }E_{\alpha }}{1+{\left(i\omega \right)}^{\alpha }\frac{E_{\alpha }}{E_{2}}}$
SLS	E, *η*, *α*	$\sigma (t)+(\frac{\eta }{E_{2}})\frac{d\sigma (t)}{dt}=E_{1}\;\varepsilon (t)+\left(\frac{\eta (E_{1}+E_{2})}{E_{2}}\right)\frac{d\varepsilon (t)}{dt}$	$E^{*}(w)=\frac{E_{1}+(i\omega )\eta \frac{\left(E_{1}+E_{2}\right)}{E_{2}}}{1+(i\omega )\frac{\eta }{E_{2}}}$
FD-SLS	E, *η*, *α*	$\sigma (t)=E_{1}+E_{2}F_{\alpha }(-\frac{E_{2}}{E_{\alpha }}t^{\alpha })$	$E^{*}(w)=\frac{E_{1}+{\left(i\omega \right)}^{\alpha }E_{\alpha }\frac{\left(E_{1}+E_{2}\right)}{e_{2}}}{1+{\left(i\omega \right)}^{\alpha }\frac{E_{\alpha }}{E_{2}}}$
Spring-port	*η*, *α*	$\sigma (t)=\eta d^{\alpha }[\varepsilon (t)]$	$E^{*}(w)=\eta {\left(i\omega \right)}^{\alpha }$

### 1.2 Viscosity and elasticity in early-stage soft tissue diseases

Viscosity and elasticity play interconnected roles in the context of early-stage soft tissue diseases. In inflammation, the Viscosity of soft tissues may increase due to changes in fluid dynamics and the presence of inflammatory mediators [18]. This altered Viscosity can impact the accurate measurement of tissue elasticity. For instance, In the liver tissue of humans, the presence of both fat accumulation (>5%) and inflammation is referred to as hepatic steatosis, commonly associated with non-alcoholic fatty liver disease (NAFLD) [31]. Contributory factors to the onset of fat accumulation and inflammation in the liver include obesity, poor dietary habits, insulin resistance, type 2 diabetes, and high sugar intake [35, 36]. This fat accumulation induces significant alterations in the viscoelastic properties of the liver tissue. Understanding and characterizing these conditions is crucial, as the early detection of liver disease provides an opportunity for timely interventions and therapies to mitigate or reverse pathological changes [10, 30]. Without early intervention, the buildup of fat in liver cells may progress to necrosis and inflammation, further contributing to the advancement of non-alcoholic steatohepatitis (NASH) and Liver fibrosis [30, 37]. As inflammation becomes apparent, there is an augmentation in both intracellular and extracellular fluid. However, the overall volume within the liver capsule is constrained. This results in a decrease in the spaces between cells and some blood vessels, contributing to the overall effect of constriction in the vascular bed and stiffening of the stress-strain response of the liver. Likewise, in the plantar tissue, early-stage inflammation related to conditions like plantar fasciitis or Heel Fat Pad Syndrome may result in subtle changes in Viscosity and elasticity that are challenging to distinguish [38]. Furthermore, in the case of prostate tumors and breast lesions, the growth of the tumor and its progression into adjacent (healthy) tissue can be viewed as an interruption of the usual balance in tissue equilibrium [39]. In both the breast and prostate, this phenomenon is described as a stromal reaction marked by the deposition of collagen to enhance the invasiveness of the tumor [39–41]. The augmentation of collagen deposition contributes to an elevation in the stiffness of the respective tissues [30].

### 1.3 Role of rheological models in tissue characterization

Rheological models are employed as essential tools to capture the change in mechanical properties of soft tissue, aiding in understanding normal, steatosis, and inflamed states of the tissue [3, 42]. Soft tissues often exhibit a power-law behavior in stiffness, reflecting the complex microstructure and composition. This power-law relationship signifies a non-linear correlation between stress and strain, mirroring the hierarchical organization of tissues across different scales [33]. The mechanical attributes of normal, steatosis, or inflamed soft tissues arise from interactions among components like collagen, fat, elastin, cells, and the extracellular matrix. At the microscale, the arrangement and density of these components play a pivotal role in determining the best rheological model for quantifying its overall tissue viscoelastic parameters [30, 42]. Several studies have supported using the Kelvin-Voigt and Maxwell fractional derivative models to measure the homogeneity of normal soft tissue [16, 30].

Moreover, K J Parker et al. employed a Composite model to characterize viscoelastic properties of soft tissue related to fat accumulation (steatosis) regardless of its limitation [30, 42]. Also, studies revealed that the progression of inflammation into soft tissue is linked to an increase in intracellular and extracellular fluid, resulting in crowding effects that reduce the dimensional spaces between cells and some blood vessels, leading to a drop-in fluid flow. Studies have examined the adjustment in fluid flow within the tissue using the microchannel flow model. K J Parker et al. utilize the Microchannel flow model to characterize Non-alcoholic fatty liver disease and observe that the Microchannel flow model can predict changes in shear modulus and shear wave speed from inflamed tissue. An advantageous feature of the fractional derivative and microchannel flow models is their ability to exhibit power-law behavior characteristics in temporal response, a phenomenon observed in various biological materials [33]. KVFD and Maxwell-FD represent 3-parameter rheological models, as shown in Table 1, but the number of parameters can be reduced to 2 depending on the specific material behavior. The parameter *a*, called fractional order in the constitutive equation of the fractional derivative model, quantifies the fractional order of the spring-pot element: when α→ 0, the fractional unit behaves like a Hookean spring (Pure elastic); when α → 1, it behaves as a Newtonian dashpot (Pure viscous). For intermediate values of α, it acts as a viscoelastic material, a characteristic common in most biological tissues [16, 24]. To our knowledge, existing reviews highlight the effectiveness of elastic parameters in characterizing tissue stiffness associated with the progression of inflammation across various tissues. They rely primarily on elastic parameters as biomarkers for inflammation using the direct elastography method [43–45]. Naotaka Nitta et al. demonstrate that the elastic parameter estimation assumes soft tissue behaves as an isotropic linear elastic body. However, typical features that deviate from this assumption include viscoelasticity, nonlinearity, and anisotropy of soft tissues. Therefore, a review by Naotaka Nitta et al. elaborated on these factors, detailing how they influence the interpretation of Shear Wave Speed (SWS) and the estimation of the elastic modulus.

Additionally, Guillermo Rus’s review discussed the role of tissue microstructure in linear mechanics, highlighting how the biological components are structured to exhibit viscoelastic and nonlinear properties within a normal and tissue with pathologies [46]. Although we briefly mention these aspects in certain sections of our review, they are crucial for the reader to understand the comprehensive scope of this review. Therefore, this review does not aim to explore various elastography techniques or address the viscosity components of tissue pathology. Instead, it focuses on the ability of parameters such as elasticity, viscosity, and time constant to stage diseases, particularly in the liver, plantar, and breast tissues. Despite numerous methods used to characterize tissue inflammation using these parameters quantitatively, researchers have not established a standard value to distinguish healthy tissue from inflamed tissue. This review includes studies utilizing mechanical rheological models to quantify these parameters. Therefore, it examines the effectiveness of existing mechanical rheological models in characterizing tissue inflammation, highlighting the lack of consensus on these models within the clinical and elastography community. The specific objective includes:

1. To review the association between elasticity and Viscosity in diseases, specifically as tissue inflammation progresses.
2. To systematically review the utilization of different Mechanical Rheological Models to characterize quantitative viscoelastic parameters of normal and stages of inflamed tissues.
3. To suggest future research directions.

## 2. Methods

We used standard systematic review methods, including search methods, inclusion and exclusion criteria, data collection, and quality assessment, based on Preferred Reporting Items for Systematic Reviews Analysis and Metal Analysis (PRISMA) guidelines [47]. The details of our method are defined below:

### 2.1 Search strategy and study selection

The authors performed the systematic review by searching two trusted sources covering various subject areas: Web of Science, Scopus, and PubMed database, mainly covering life sciences and Biomedical literature. The authors reviewed only English publications with keywords. The literature search was conducted on January 2024, allowing publication from January 2000 to January 2024 using the following terms: (("Elasticity" OR "Viscosity" OR "Viscoelastic*") AND ("Liver" OR "Plantar" OR "Breast" OR "Prostate" OR "Thyroid" OR "Kidney" OR "Bladder") AND ("Shear wave" OR "Shear wave dispersion" OR "Time of flight") AND ("Ultrasound Elastography")) OR (("Elasticity" OR "Viscosity" OR "Viscoelastic*") AND ("Liver" OR "Plantar" OR "Breast" OR "Prostate" OR "Thyroid" OR "Kidney" OR "Bladder") AND ("Shear wave" OR "Shear wave dispersion" OR "Time of flight") AND ("Rheological models"))

We employed Zotero 6.0.35 to remove duplicates of articles retrieved from three databases (Web of Sciences, Scopus, and PubMed). The search results were downloaded in CSV format and then imported into Zotero. We consolidated all imported references into a single collection within Zotero to facilitate duplicate detection and management. Zotero interface “Duplicate Items” displayed all references identified as duplicates. We reviewed the details for each set of duplicate references to ensure accuracy. Zotero provides a side-by-side comparison of duplicates, highlighting differences. We then used the “Merge Items” function to combine duplicates into a single reference, ensuring no loss of critical information. Then, the authors screened studies by title, abstract, and full-text screening in Rayyan (http://rayyan.qcri.org/) for the final selection. Two independent reviewers (JJK and RRS) conducted a screening process in Rayyan when the BLIND function was ON. We also performed a manual search from the reference list of each relevant article. The authors resolved the discrepancies in the studies upon selection through discussion and agreement following independent appraisal by another author (ZW).

### 2.2 Inclusion and exclusion criteria

**Fig 2:**
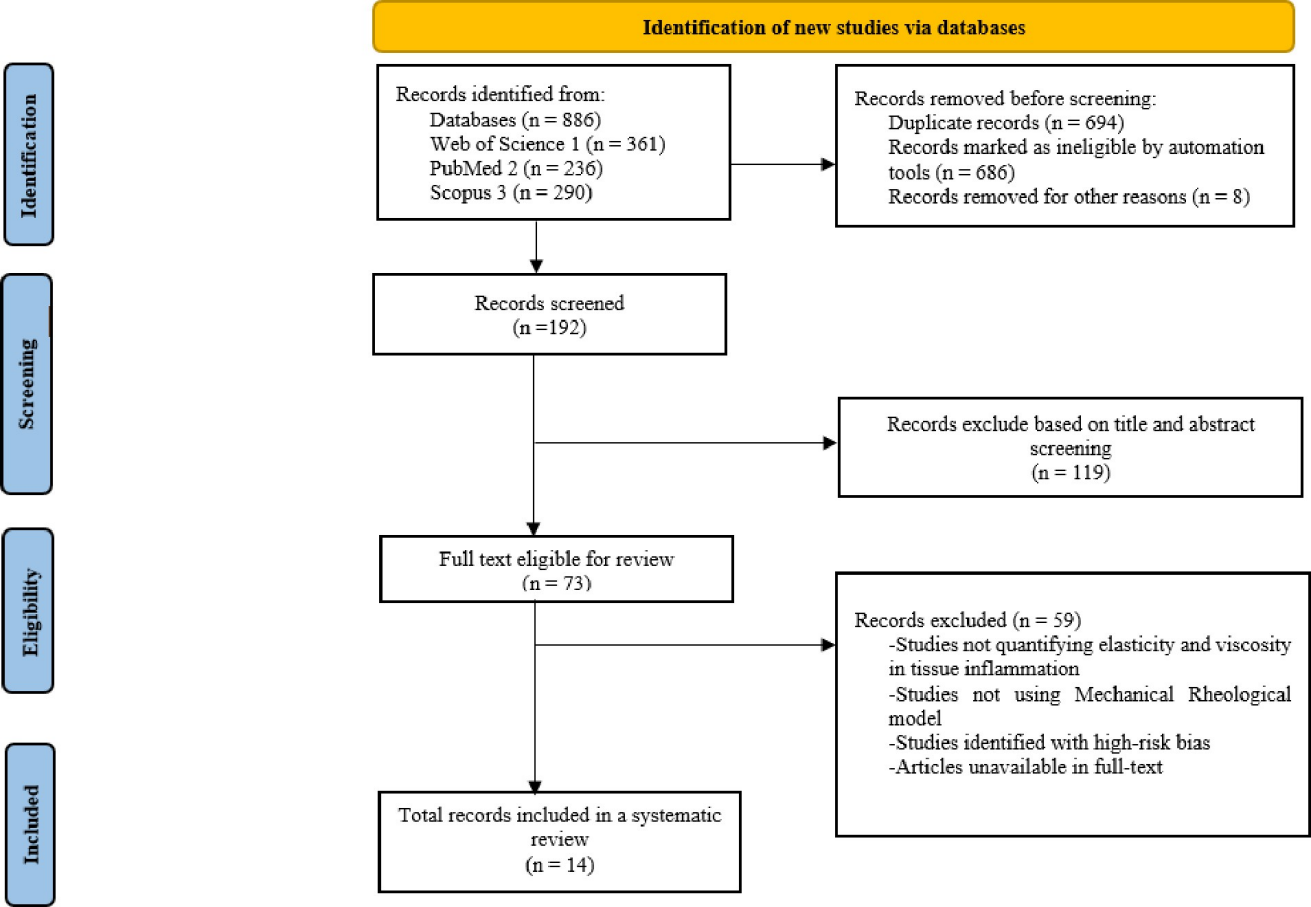
PRISMA flowchart of the studies through the review.

The criteria for the inclusion of the searched articles in the review were: (i) articles that describe the use of Ultrasound Shear wave Elastography, (ii) studies that reported on the use of Mechanical Rheological model (e.g., Maxwell, KV, SL, SLS, and KVFD) for Quantifying viscoelastic properties of the tissue. (iii) All studies investigating the role of elasticity and viscosity in detecting tissue inflammation and early-stage soft tissue diseases. (iv) All studies that quantify viscoelastic parameters in vivo /ex-vivo from human tissue or mimic phantom. The articles searched underwent the following exclusion criteria to ensure a relevant selection of studies. We excluded studies that report other modalities of tissue characterization (e.g., Magnetic resonance elastography-MRE) and those that did not report on the use of the Mechanical Rheological model for quantifying the viscoelastic properties of tissue. We also excluded non-English studies due to language translation limitations and potential biases. We omitted articles unavailable in full text to guarantee comprehensive data extraction and analysis. To maintain the significance of our research questions, we excluded studies that did not address the critical parameters of elasticity and Viscosity in tissue inflammation. We focused on original research data, excluding reviews, editorials, opinion pieces, and conference abstracts. Based on our quality assessment criteria, we excluded studies with a high risk of bias to ensure the reliability and validity of our findings. We excluded publications before 2000 to emphasize recent and appropriate research. Lastly, we excluded studies that did not involve human subjects or relevant tissue models, as they did not align with our research focus. We used the PRISMA literature search checklist to present the final full article results, as shown in Fig 2 below.

### 2.3 Data items

We extracted the following data and results from the full-text articles identified for inclusion in this review: (i) For a comparative analysis of the viscoelastic model, the author collected these items: publication year, authors, tested model, Shear wave speed test method used, Frequency, vivo/ex-vivo experiment and Suggested best model. (ii) The author collected these items for mechanical insights into disease associations: publication year, author, technique, tissue name, elastic coefficient, Viscosity, Frequency, and model used.

### 2.4 Data extraction

After duplicate checking and screening of all the articles according to the inclusion and exclusion criteria, the same two reviewers (JJK and RRS) extracted the data from the appropriate articles and assessed the risk of bias. Differences in the extracted data were resolved by agreement between the reviewers. We conducted data extraction using the typset.io program and through manual extraction by reading the full article.

### 2.5 Risk of bias assessment

Two researchers (JJK and RRS) independently assessed the risk of bias in identified studies using the risk of bias tool from the Cochrane Collaboration [48]. The assessment tool evaluates the following: randomization process, deviations from intended intervention, missing outcome data, measurement of outcome data, and selection of reported results. Each item could be classified as high, low, or some concerns. When there was insufficient information to determine whether a study met the scoring criteria, it was rated as having some concerns. The researchers resolved any discrepancies in their judgments through discussion.

## 3. Results

### 3.1 Map visualization

**Fig 3:**
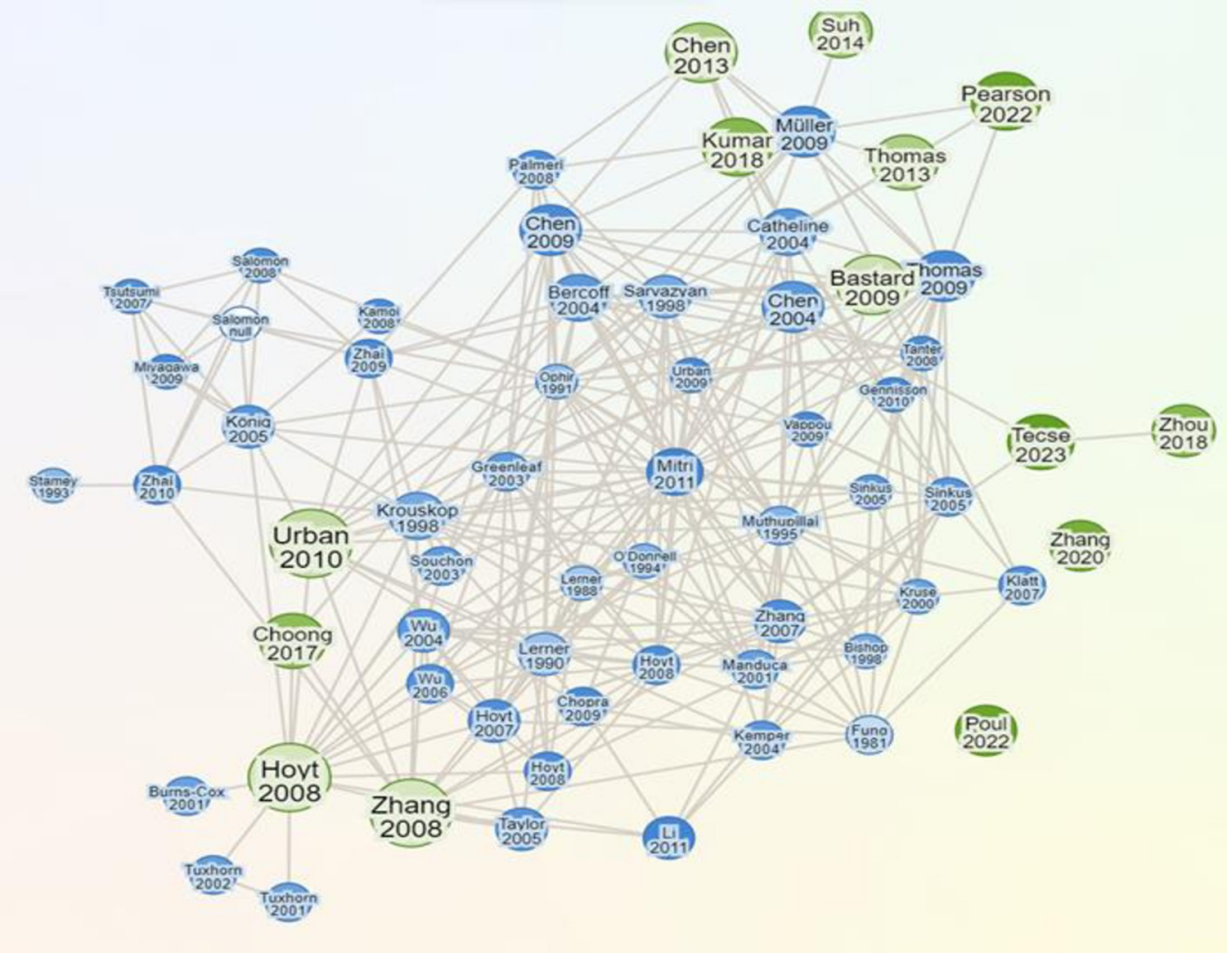
Illustrate the network view based on the similarity of papers closely related to our selected references and most cited publications on the Visualization map.

The Research Rabbit program generated the maps based on network data for this review. The program is an AI-powered tool for finding academic publications developed in 2021. It effectively examines the relationships between existing seeds and candidates’ publications. The tool utilizes visualization maps that list earlier, later, and comparable articles and assists in discovering publications connected to one or more seed papers. Fig 3 shows the relationships between existing seed papers and candidate-selected papers visualized as nodes in a network. The similarity map reveals the interdisciplinary relevance of the reference papers. This interdisciplinary relevance indicates that the reference papers contribute to a multidisciplinary understanding of applying the mechanical rheological model in assessing elasticity and viscosity in other fields. Therefore, the connection of reference papers to many other papers is essential within the field.

**Fig 4:**
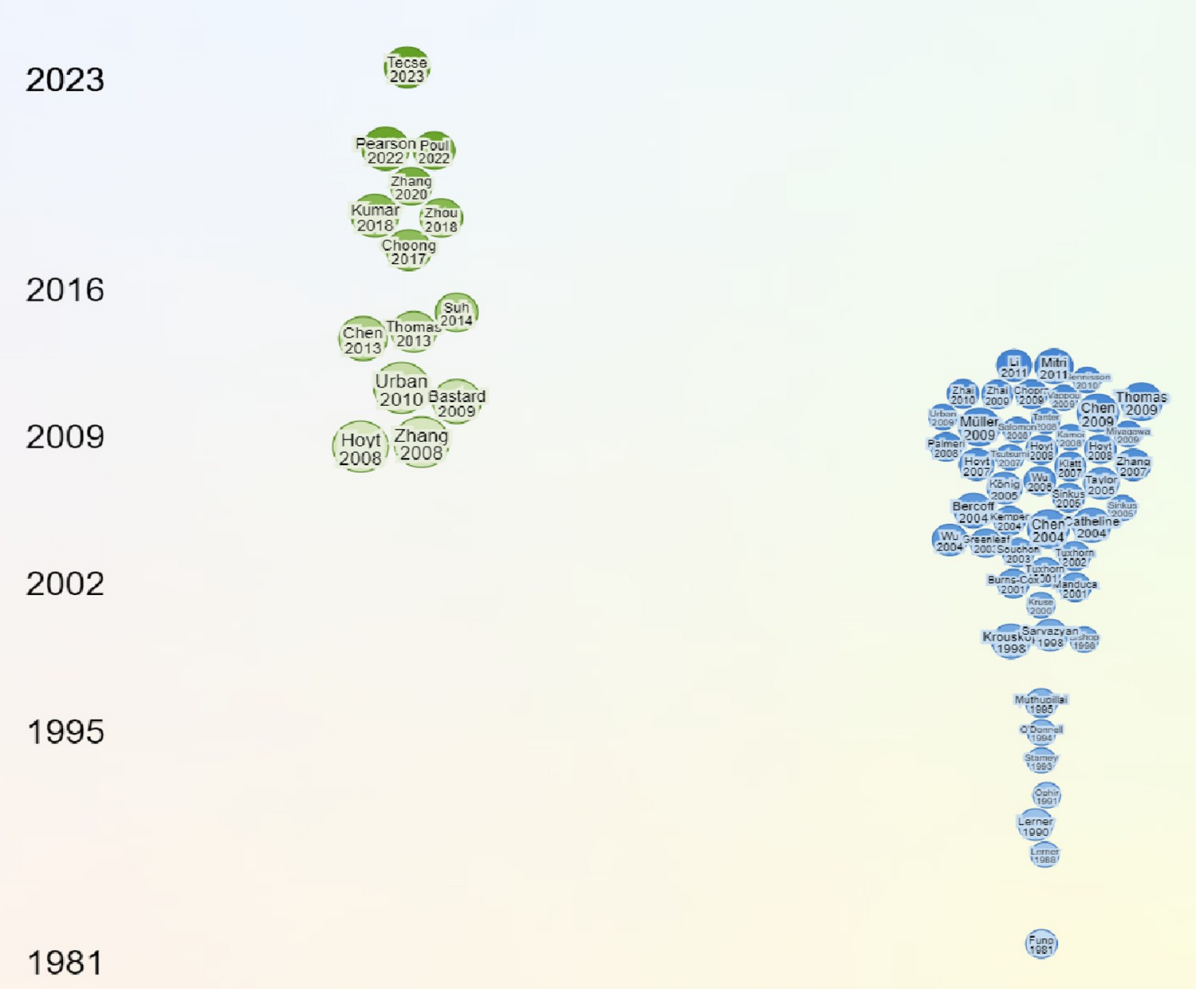
Illustrate the timeline view of related publications on the visualization map.

Fig 4 below shows a timeline perspective plot that displays the publications by year, illustrating when researchers published work in the field. The trend analysis over time shows a significant increase in publications related to assessing tissue inflammation using viscosity and elasticity starting around 2000, with a peak in recent years. This increase indicates the growing attention on applying the mechanical rheological model in assessing elasticity and viscosity in tissue inflammation, possibly driven by the increasing global application of Artificial intelligence to healthcare diagnosis. The increasing number of publications in recent years reflects a rising interest and perhaps urgency in understanding the relationship between elasticity, viscosity, and tissue inflammation. The green nodes in Figs 3 and 4 represent the existing seed publication. Meanwhile, the blue nodes show the candidate publications. The darker green nodes show the most recent publication and the larger circle shows the publication rank according to citations. The maps show that the fourteen identified studies met the criteria for being considered the most recently referenced studies. Therefore, it points to a potential new area of focus in the association between elasticity and Viscosity in tissue inflammation using different mechanical rheological models to characterize quantitative viscoelastic parameters of normal and inflamed tissues.

### 3.2 Risk of bias and quality of included studies

We evaluated the quality of the included studies using the risk of bias assessment tool for standard randomized controlled trial evidence recommended by the Cochrane Collaboration [48]. Due to the nature of the identified studies, we have considered the technique used for tissue characterization as an intervention to test the effectiveness of the viscoelastic parameter. This approach was essential for evaluating the accuracy and applicability of the rheological model in the techniques used. The quality assessment revealed that 81.8% of studies had some concerns about the randomization process, and 18.2% were at high risk, with no studies rated as low risk. Nine studies claimed to have used randomization of participants but did not provide detailed information on how they conducted randomization. They also mentioned that neither researcher who performed the measurement was aware of the participant’s condition, leading us to rate these as having some concerns. We rated two studies that did not mention randomization criteria as high-risk.

Regarding deviations from intended interventions, 81.8% of studies were at low risk, indicating minimal deviations. In comparison, 9.1% had some concerns, and another 9.1% were at high risk because some of the interventions were not associated with the rheological model to quantify the viscoelastic parameter of the soft tissues. Cecile Bastard’s research was rated high risk in this domain, while the rest did not deviate from the intended intervention. All studies received a low-risk rating in the missing outcome data domain, suggesting that missing data did not significantly bias the results as each technique could at least quantify parameters relating to the participant’s condition. For the measurement of the outcome domain, 81.8% of studies were at low risk because the nine studies ensured that the pathologists who performed biopsies on the same group did not know the results from the intervention techniques. However, 9.1% revealed some concerns, and 9.1% revealed high risk.

**Fig 5:**
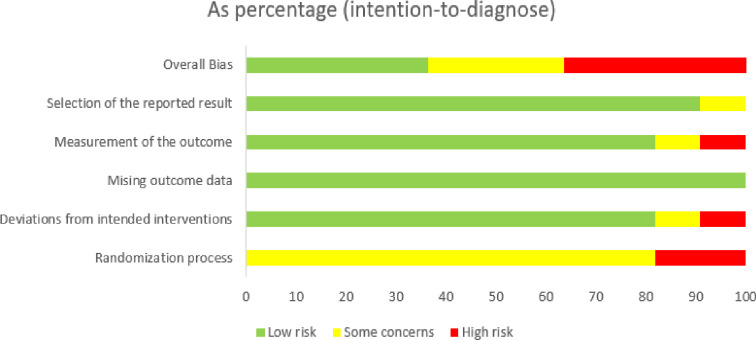
Risk of bias graph.

**Fig 6:**
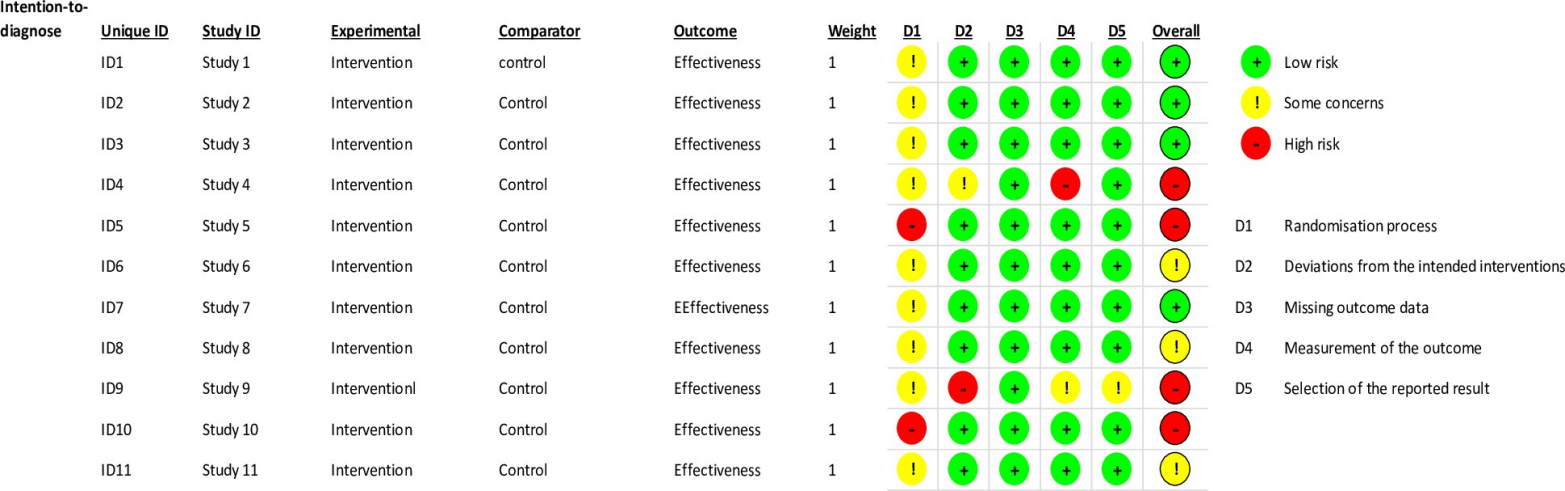
Risk of bias summary.

Regarding selecting the reported result, most studies (90.9%) were at low risk, indicating comprehensive outcome reporting, with only 9.1% having some concerns. There was no evidence of other biases, so this item was low risk. The overall bias varied, with 36.4% of studies at low risk, 27.3% with some concerns, and 36.4% at high risk. These assessments ensure that the studies in our systematic review provide reliable and valid data on the accuracy of various techniques in conjunction with mechanical rheological models in characterizing tissue inflammation. The comprehensive evaluation of the studies is illustrated in Figs 5 and 6, which provide a visual representation of the risk of bias across the included studies, and the overall quality assessment results for the included studies are summarized in Table 2, providing a comprehensive overview of the risk levels across different domains.

**Table 2:** The overall quality assessment results for all included studies.

Domain	Low risk (%)	Some Concerns (%)	High risk (%)
Randomization Process	0	81.8	18.2
Deviations from Intended Interventions	81.8	9.1	9.1
Missing Outcome Data	100	0	0
Measurement of the Outcome	81.8	9.1	9.1
Selection of the Reported Result	90.9	9.1	0
Overall Bias	36.4	27.3	36.4

### 3.3 Comparative analysis of viscoelastic models

The appropriate choice of a viscoelastic model for soft biological tissues is an area of ongoing research in ultrasound elastography [24]. The development of a universally accepted or optimal model for characterizing specific tissues, or even across diverse tissues, has not reached a consensus within the scientific community [16]. The absence of a widely agreed-upon standard hinders the establishment of a singular, comprehensive model that can accurately represent the distinctive properties of various tissues. This lack of unanimity underscores the complexity and variability inherent in tissue characteristics, necessitating further research and exploration to refine and potentially converge toward a more universally applicable model. Boran Zhou et al. compared five viscoelastic models (KV, Maxwell, SLS, spring-pot, and KVFD) through analytical and experimental studies in USWE. Boran Zhou et al. demonstrated that the KV and KVFD models exhibited superior performance among the examined models, showcasing notably low analytical residual errors of 1.406e-20 and 6.72e-13, respectively. Furthermore, the experimental phase of the study substantiated these findings, with both the KV and KVFD models demonstrating low residual errors of 1.0569 and 1.0529, respectively. Sedigheh S Poul et al. use other techniques to assess three rheological models: the KV model as a 2-parameter model, SLS, and the KVFD model as 3- parameter models. Sedigheh S Poul et al. evaluated the best model based on two criteria. Firstly, Model parameters derived from fitting the frequency domain (phase velocity dispersion) data should predict the time domain (stress relaxation) results. Conversely, parameters obtained from fitting the time domain data should predict the frequency domain results. Secondly, a model that can precisely capture a range of crucial experimental data and achieve this with minimal viscoelastic parameters. Sedigheh S Poul et al. conducted all the experiments on ex vivo bovine liver and shear wave speed test through Harmonic shear test (HST) using a rheometer.

Sedigheh S Poul et al. also utilized Reverberant shear wave (RSW-US) ultrasound and Reverberant shear wave optical coherence elastography (RSW-OC), each suitable for a specific frequency range as shown in Table 3. HST assessed the speed dispersion at low Frequency, RSW-US elastography for medium Frequency, and RSW-OC for higher Frequency. The higher frequency is out of scope for RSW-US to set because the technique is affected by increased noise and attenuation [16]. Crucially, Sedigheh S Poul et al. conducted a stress relaxation to capture the time-dependent behavior of ex vivo bovine liver tissues, yielding a stress relaxation curve in the time domain. Sedigheh S Poul et al. then fitted shear wave dispersion data from each experimental test to the dispersion relationship of three rheological models. The KV and KVFD models effectively captured shear wave speed dispersion within the middle-frequency range. However, SLS excelled in capturing this dispersion at low frequencies. Notably, the KVFD model exhibited significant performance across low and high-frequency ranges, achieving a coefficient of determination minimum (R^2^) of 0.96. The KVFD model remarkably fulfilled the first criterion compared to the Stress relaxation experimental data.

**Table 3:** Studies evaluating the best Rheological model.

Author	Tested Model	Vivo/ Ex Vivo	Sample	Shear wave speed test Methods used	Frequency Used	Suggested Best Model
[24]	KV, Maxwell	Ex Vivo	Tissue-mimicking phantom	USWE	(100,150,200,250,300) HZ	KVFD model
	SLS					
	Spring pot					
	KVFD					
[16]	KV	Ex Vivo	Bovine liver Tissue	HST	1HZ-15HZ	KVFD model
	SLS			RSW-US	200HZ-400HZ	
	KVFD			RSW-OC	1KHZ	
[50]	KV, Maxwell, SL, SLS	Ex-Vivo	Tissue-mimicking phantom	RSW-US	(450,500,550,600) Hz.	FD-Maxwell and KVFD-models
	KVFD, FD-Maxwell FD-SL, FD-SLS					

In contrast, the KV and SLS models predicted constant stress, deviating from the observed experimental data. Furthermore, concerning a contrariwise criterion, the KV model exhibited an increased shear wave speed (SWS), the SLS model showed negligible SWS dispersion, and the KVFD model reasonably predicted the frequency domain behavior compared to experimental data. Sedigheh S. Poul et al. demonstrated the KVFD model as the best model as it consists of 3-parameters (Young’s modulus *E*_0_, fractional order of the spring-pot element *a* and dashpot viscosity η), which can be reduced 2-parameter for very soft viscoelastic media when *E*_0_ is negligible [16, 21, 49]. Romero et al. conducted a study on healthy volunteers with no history of foot pathologies to assess the reliability of reverberant shear wave elastography (RSWE) in measuring shear wave speed (SWS) in the plantar soft tissue. Aldo Tecse et al. replicated the phantom using the SWS data obtained from Romero et al.’s study and explored the most suitable rheological model among the eight available models. Sedigheh S. Poul et al.’s approach verified the best. KVFD and FD-Maxwell models effectively replicated the viscoelastic behavior of plantar soft tissue using a minimal number of parameters, achieving a coefficient of determination value (R^2^) of 0.72 for both models. Results aligned with [14, 29], indicating that the KV, Maxwell, and SLS models inadequately describe soft tissue’s time domain (SR) behaviors.

### 3.4 Elasticity, viscosity, inflammation: Disease correlations

The biomechanical properties of different tissues in correlation with inflammatory diseases are presented in Table 4.

**Table 4:** Studies employed viscoelastic parameters to characterize various soft.

Author	Technique	Tissue Name	Normal tissue	Inflamed tissue	Elastic coefficient (Pa)	Viscosity	Fractional order derivative	Frequency Used (Hz)	Model used	Normal and inflamed tissue				
										Parameter	Sensitivity 95% CI	Specificity 95% CI	P-Value	AUROC (95% CI
[8]	Fibro Scan & Spectroscopy algorithm	Liver	Yes	No	At 50Hz -1091 At 75Hz-1064	At 50Hz -1091 Pa-s At 75Hz-1064 Pa-s	N/A	50,75	Voigt	E	At 50Hz—50 At 75Hz- 60.0	At 50Hz -77.3 At 75Hz- 45.5	At 50Hz—0.537 At 75Hz- 1	At 50Hz—0.557 At 75Hz—0.5
			No	Yes	At 50Hz -999 At 75Hz-1077	At 50Hz -3.0 Pa-s At 75Hz-2.3 Pa-s		50,75		η	At 50Hz -70.0 At 75Hz- 65.0	At 50Hz -90.9 At 75Hz- 77.3	At 50Hz—<0.001 At 75Hz—0.002	At 50Hz—0.814 At 75Hz—0.784
[55]	Fibro Scan & SSI		No	Yes	F1: 6.8 ± 700 F2: 9.8 ± 1600 F3:11 .3 ± 1700 F4: 22.3 ± 2100	N/A	N/A	50,150	Voigt	E	N/A	N/A	N/A	F0>F1- 0.75 F0>F2–0.79 F0>F3–0.79 F0>F4—N/A
										η	N/A	N/A	N/A	F0>F1–0.76 F0>F2–0.77 F0>F3–0.82 F0>F4—N/A
[13]	SDUV		Yes	No	4650	2.97 Pa. s	N/A	95,190 285,380	Voigt	E	1	0.87	p<0.05	0.98
										η	1	0.63		0.86
[56]	SWE		Yes	No	2600–6200	N/A	N/A	N/A	N/A	E	91.00%	95.90%	p<0.05	N/A
[57]	SWE		Yes	No	6630±16.5	N/A	N/A	N/A	N/A	μ	1	1	p<0.05	N/A
			No	Yes	51450± 14.96									
					49890±13.82									
[58]	VCTE		Yes	No	3300±0.6	1.8 ± 1.0 Pa. s 1.9 ± 0.7 Pa. s	N/A	20–250	Voigt	N/A	N/A	N/A	N/A	N/A
[59]	RS-SWE	Plantar tissue	Yes	No	576 00(37.8–119.7)	1.06 (0.86–2.00) Pa-s	0.4 (0.37–0.48)	450,500 550,600	FD	N/A	N/A	N/A	N/A	N/A
			Yes	No	1e-11 (9e-12–24.99)	2.56 (0.94–4.64) Pa-s	0.2684 (0.19–0.51)		KVFD					
[60]	SDUV	Breast	Yes	No	10200±0.97	(1.41±0.67) Pa-s	N/A	50–400	Voigt	E	N/A	N/A	N/A	N/A
			No	Yes	(14000±1.12)	(2.83±1.47) Pa-s.				η			P = 0.000125	
[61]	AFM		Yes	No	655.828 ± 223.417	5.959 ± 1.157 s	0.654 ± 0.100	N/A	KVFD	E	N/A	N/A	p = 0.007	N/A
			No	Yes	729.457 ± 345.236	6.292 ± 1.875 s	0.662 ± 0.085			τ			p = 0.045	
[49]	Stress relaxation	Prostate	Yes	No	15900 ± 5.9	3.61 ± 1.25 kPa s^α	0.2154 ± 0.0417	150	KVFD	N/A	N/A	N/A	N/A	N/A
			No	Yes	40400 ± 15.7	8.65 ± 3.40 kPa s^α	0.2247 ± 0.0304							
[21]	Stress relaxation	Prostate	Yes	No	3800 ± 1.8	3.6 ± 1.3 kPa s^α	0.22 ± 0.04	0.1–150	KVFD	N/A	N/A	N/A	N/A	N/A
			No	Yes	7800 ± 3.3	8.7 ± 3.4 kPa s^α	0.23 ± 0.03							
[62]	SDUV	Prostate	Yes	No	5200±2.55	2.68±1.47 Pa s	N/A	(50–400)	N/A	N/A	N/A	N/A	N/A	N/A

#### 3.4.1 Plantar tissue

Plantar tissues, encompassing muscles, tendons, ligaments, and the plantar fascia, play a pivotal role in the intricate biomechanics of the foot [38, 50]. The plantar fascia, a robust band of connective tissue, extends along the sole, connecting the heel bone to the toes. This crucial structure is integral to the overall support and flexibility of the foot. Correlating with the plantar tissues, the heel pad, a specialized area beneath the heel bone, complements this dynamic system. The heel pad comprises two primary layers: the microchamber layer, located on the surface, and the macro chamber layer, situated deeper [51]. The microchamber layer exhibits higher tissue stiffness, with an elastic modulus approximately ten times greater than the macro chamber layer [38]. Within the heel pad, the heel fat pad serves as a cushion, absorbing shock and dispersing plantar force during walking, particularly in its anterior internal portion containing a fat lump [52]. The viscoelastic properties of the heel fat pad (HFP) can serve as a mechanical biomarker for normal plantar tissue and characterize inflamed plantar fascia (plantar fasciitis) [50]. It is crucial to differentiate between these conditions. Heel Fat Pad Syndrome (HFPS) emerges as a distinct pathology contributing to plantar heel pain, the second leading cause after plantar fasciitis [53, 54]. Aldo Tecse et al.’s findings, indicate that fractional derivative models KVFD and FD-Maxwell effectively characterize the mechanical behaviors of plantar soft tissue in healthy volunteers, encompassing Viscosity and elasticity, as shown in Table 4.

#### 3.4.2 Liver tissue

Fibrosis staging is crucial for assessing disease progression and determining appropriate treatment. In the examination of liver fibrosis, the study by Thomas Deffieux has yielded convincing results regarding the utility of Young’s modulus and Viscosity as key biomechanical parameters. The findings underline the reliability of Young’s modulus as an effective estimator of the fibrosis stage, offering a valuable noninvasive path for assessment. Moreover, Deffieux’s investigation reveals the significance of Viscosity in predicting the fibrosis stage, showcasing distinct trends at frequencies of 50Hz and 150Hz.

These nuanced frequency-dependent variations in Viscosity provide a novel perspective, enhancing the understanding of liver tissue mechanics. Deffieux assessed Viscosity by estimating the shear wave dispersion curve from the data set through FibroScan & Supersonic shear imaging (SSI) and fitting Voigt’s model.

Model selection criteria were because of its few parameters. Arthur Pearson et al. developed an algorithm based on the KV model to extract viscoelastic properties (i.e., ηVoigt and μVoigt) of healthy volunteers and patients with steatosis without severe fibrosis. Arthur Pearson et al. aimed to noninvasively assess liver steatosis using a FibroScan and an algorithm based on the Voigt rheological model. Arthur Pearson et al. compared the Fibroscan default values (μFibroscan) of liver stiffness with viscoelastic parameters from the developed algorithm to find their correlation. ηVoigt and μFibroscan were well correlated for the 50 Hz and 75 Hz excitations (r = 0.75 and r = 0.62), respectively. On the other hand, μVoigt doesn’t correlate with μFibroscan for both excitations. Upon evaluation of ηVoigt and μFibroscan values in healthy volunteers and patients with steatosis, both values were significantly higher in patients with liver steatosis compared to healthy volunteers, and μVoigt was not entirely different between the two groups as shown in Table 4

Shigao Chen et al. conducted a study to explore whether the viscosity values obtained through a viscoelastic model could enhance the accuracy of fibrosis staging. They utilized ultrasound radiation force to induce shear waves and employed a push pulse with a frequency of 2MHz to generate shear dispersive waves. The shear wave dispersion ultrasound vibrometry (SDUV) technique analyzes the ultrasound data. This technique measured elasticity and Viscosity by evaluating shear wave propagation speed dispersion. He used the time-to-peak (TTP) method, neglecting tissue viscosity and estimating only adequate elasticity from the effective shear wave speed. The study used shear wave components with 95Hz, 190Hz, 285Hz, and 380Hz to estimate shear wave speed.

Shigao Chen et al. explicitly mentioned that no rheological model has been universally agreed upon as the best for describing the response of soft tissues. Shigao Chen et al. selected the KV model based on previous studies that identified it as the most suitable [63]. Furthermore, Shigao Chen et al. decided it based on the frequency range used by the SDUV technique, as they considered the KV model more effective than others.

The study’s findings indicated that Voigt elasticity and Viscosity measured with SDUV and effective elasticity measured with TTP increased with advancing liver fibrosis stage. The degree of fibrosis showed a correlation with Voigt elasticity (r = 0.83), Voigt viscosity (r = 0.68), and effective elasticity (r = 0.81). The area under the ROC curve for distinguishing between grade F0-F1 fibrosis and grade F2-F4 fibrosis was 0.98 for elasticity measured with SDUV, 0.86 for Viscosity estimated with SDUV, and 0.95 for elasticity measured with TTP. Based on these observations, the suggested optimal cutoff values were 4.65 kPa for Voigt elasticity measured with SDUV and 2.97 Pa. s for Voigt viscosity measured with SDUV. While the study indicated a negligible viscosity contribution in characterizing liver fibrosis, researchers still regard it as a valuable parameter in assessing it. The current studies suggest conducting additional investigations using an enhanced rheological model [13].

#### 3.4.3 Breast tissue

The soft tissue components of the breast encompass cells and the extracellular matrix (ECM) [64, 65]. Studies indicate that the biomechanical and structural characteristics of both the cell and ECM differ across stages, including normal tissue, benign focal breast lesions (inflammatory or hyperplastic), and malignant focal breast lesions (ductal carcinoma in situ (DCIS) and invasive ductal carcinoma (IDC) [66]. Throughout the progression of the disease, the stromal reaction leads to increased collagen and fibronectin density, reinforcing cell adhesions. Simultaneously, the desmoplastic reaction increases fibrin and fibronectin levels, increasing Viscosity [66]. Hongmei Zhang et al. conducted a study employing a KVFD model in the time domain to distinguish between normal, benign, and malignant tissues at a cellular-tissue level. The viscoelastic parameters significantly correlated with the histopathological features of the breast samples. The KVFD model accurately fitted all of the force-indentation data obtained by indentation-type atomic force microscopy (IT-AFM) with an R^2^ > 0.95. Hongmei Zhang’s study unveiled promising findings, indicating notable distinctions in elastic modulus, Viscosity time constant, and fluidity among different tissue groups. The results suggested that the Elastic modulus and fluidity (E_0_, α) of the KVFD model can serve as viscoelastic biomarkers for diagnosing normal, benign, and malignant breast tissues. He noted a significant difference (p < 0.007) in Elastic modulus between every pair of sampled groups. Additionally, essential distinctions Hongmei Zhang et al. observed between normal and malignant (p < 0.0001) as well as benign and malignant (p < 0.0001) groups based on fluidity α, with no notable difference between normal and benign groups (p = 0.057). Furthermore, there were significant variations between normal and benign (p = 0.045) and normal and malignant (p = 0.009) groups concerning measurements of the viscous time constant τ. However, Hongmei Zhang et al. identified no significant difference between the benign and malignant groups (p = 0.203). Viksit Kumar conducted a similar study using the SUDV technique in vivo to distinguish between breast masses. This time, the KV model was applied in the frequency domain to assess the viscoelastic properties of the breast masses, leveraging the frequency dispersion exhibited by breast tissue. The estimated viscoelastic parameters as shown in Table 4 effectively differentiated between benign, malignant, and normal tissues, with P-values below 0.05. Shear elasticity did not significantly distinguish between normal and benign masses. Viksit Kumar reported a significant difference in shear Viscosity between malignant and benign tissues, malignant and normal tissues, and benign and normal tissues (p = 4.13 × 10–5, 3.67 × 10–7, and 1.25 × 10–4, respectively). In simpler terms, shear Viscosity could differentiate between normal and benign tissue.

## 4. Discussion

Deliberation from different studies suggested that the criteria for selecting the best tissue characterization model should rely on a few parameters[16, 24, 59]. Hence, many studies have used the KV model because it has fewer parameters and has shown good performance in other studies[8, 13, 20, 55, 58, 66]. Moreover, a model with few parameters reduces computation because of its lower complexity [67]. The above factor has been considered vital, but selecting the model relying on how soft tissue responds to mechanical deformation is more critical than depending only upon a few parameters. The response of the normal tissue and pathological tissue upon application of deformation is different [8, 21, 61]. Some tissues, like the liver, are highly dispersive, and many pathological tissues are also dispersive [8, 16]. The finding by Sedigheh S. Poul et al. revealed that the KV model produces negligible dispersion at low Frequency but fits well in the mid-range Frequency (200Hz-400Hz). Moreover, the SLS model behaves very well at low Frequency (1HZ-15HZ) but exhibits inaccuracy at high Frequency [27]. With fractional derivative models like KVFD and FD-Maxwell, the observation looks different among different studies [24, 27, 50]. KVFD performs well regarding goodness of fit over the wide range of frequencies as shown in Table 3 and employs the fewest number of significant fitting parameters [27]. In addition, both KVFD and FD-Maxwell demonstrated a more efficient ability to mimic the viscoelastic properties of the plantar soft tissue across a broad frequency range (450Hz, 500Hz, 550Hz, 600Hz) using a minimal set of model parameters. Aldo Tecse et al. achieved an R^2^ value of 0.72 for both models. Moreover, fractional derivative models have demonstrated a strong correlation between experimental data and model predictions. Man Zhang et al. offered the effectiveness of the KVFD model for modeling the stress relaxation responses of normal and cancerous prostate tissues. The R^2^ value was > 0.9747, of which Man Zhang et al. used the same relaxation data for fitting the KV model, and the R^2^ value was not significant (r<0.4) [49]. With that observation from rheological models, several clinical studies have sought to evaluate the role of rheological models’ elasticity, Viscosity, and other parameters in assessing disease staging within soft tissue. Deffieux et al. underwent a clinical study using the Fibro Scan & SSI technique. They included 120 patients with chronic liver diseases to evaluate the applicability of viscoelastic properties in characterizing fibrosis, steatosis, and activity staging. METAVIR scoring system was used by Deffieux et al. to assess fibrosis and inflammation. His ROC analysis of each parameter shows that Voigt elasticity and Viscosity were less efficient in staging liver fibrosis as shown in Table 4. Moreover, Viscosity showed an insignificant correlation with either steatosis or fibrosis. A similar study by Shigao Chen et al. that included 45 patients with a means of SUDV revealed inefficient Viscosity in evaluating fibrosis. However, in contrast with Deffieux et al. & Shigao Chen et al. studies, Voigt Viscosity showed higher significance in predicting steatosis in the study by Arthur Pearson et al. Voigt viscosity [AUROC = 0.814 and 0.784 for 50 Hz excitation and 75 Hz excitation (95% CI: 0.680–0.947 and 0.642–0.926), respectively] was able to identify patients with and without steatosis, having sensitivity 70% and 65% and specificity of 90.9% and 77.3% at 50Hz and 75Hz excitation respectively [8]. The variation of results to the former study of Deffieux et al. & Shigao Chen et al. could be attributed to variation of etiology among recruited subjects. Liver stiffness relies on the etiology, such as Non-alcoholic steatohepatitis or hepatitis C virus infection, even when the fibrosis level is the same [19]. Therefore, Arthur Pearson et al. recruited subjects with a single etiology (NAFLD), which might lead to a different result [8].

Additionally, other studies focused mainly on utilizing SWE to assess one parameter: elasticity [56, 57]. Kah Lai Choong was able to show that the mean (SD) elasticity value for normal liver tissue obtained with SWE is (6.6 [1.7] kPa. The study explored the role of SWE in characterizing focal liver lesions, including hepatocellular carcinoma and metastases, based on tissue elasticity, proposing a diagnostic cutoff value for differentiating malignant lesions from normal liver tissue. A similar study by Chong Hyun Suh focused on establishing normal reference range values for hepatic elasticity (2.6–6.2 kPa) using (SWE) in patients with potential living-donor liver transplantation candidacy. 6.2kPa was suggested as the cutoff value for normal liver tissue, though his findings included subjects with simple hepatic steatosis. Earlier research employing acoustic radiation force impulse and transient elastography methods demonstrated that the accuracy of measuring liver elasticity may be compromised by BMI and the presence of a thicker layer of subcutaneous fat around the liver (Steatosis) because the fat surrounding the liver has different acoustic characteristics and tissue composition compared to liver parenchyma[14, 68]. The interfaces within fatty tissue may alter the pattern of shear waves, impacting measurement accuracy [2, 30].

Additionally, liver stiffness values may be elevated due to active liver inflammation. Hepatic necroinflammation, characterized by the accumulation of extracellular matrix proteins and the formation of hepatic fibrosis with abnormal collagen deposition, can alter the architecture of the liver, potentially leading to falsely elevated values in liver stiffness measurements [69]. Surprisingly, [65] and [66] discovered that steatosis and BMI did not significantly affect normal liver elasticity measured with SWE.

In clinical application, some techniques in Table 4 have deployed shear wave dispersion in conjunction with a rheological model to study the effect of viscoelastic parameters in characterizing normal and pathological tissue. VCTE is the best method to characterize liver stiffness [8]. Yet, in a study by Arthur Pearson et al., he estimated shear elasticity (μVoigt) and shear Viscosity (ηVoigt) from the data acquired by fibroscan (VCTE) using the KV model. Among the parameters he evaluated, shear Viscosity (ηVoigt) and Fibroscan default value (μFibroscan) of liver stiffness were observed to be higher in patients with steatosis, and shear elasticity was still the same in both healthy and patients with steatosis. This observation revealed the significance of Viscosity in characterizing soft tissue with pathologies related to fat accumulation and the discrepancy of the VCTE technique. Moreover, as VCTE ignores the effect of dispersion, it may lead to overestimation of liver stiffness in patients with obesity, which is why VCTE identified advanced fibrosis. At the same time, the biopsy showed minimal fibrosis in a study by [14].

Several other researchers have employed Shear Dispersion Ultrasound Vibrometry (SDUV) techniques to differentiate between normal and pathological tissues in both the breast and prostate [23, 60]. Based on their findings, the estimated elasticity and viscosity parameters, derived by fitting the shear wave dispersion into a rheological model, were insignificant in distinguishing between normal and benign breast lesions. However, they introduced the time constant parameter(τ), a dependent variable for both elasticity and Viscosity, to assess its potential significance in differentiating between normal and benign lesions. Hongmei Zhang et al. found significant differences between normal versus benign (p = 0.045) and normal versus malignant (p = 0.009) groups based on KVFD measurements of τ. Still, Hongmei Zhang et al. found no significant difference between the benign versus malignant groups (p = 0.203). Compared with the study by Visit Kumar et al., the variance in measurements of Voigt τ of malignant masses was smaller than benign masses and normal tissue. Normal tissue had a high variance in τ, and values overlapped with those of the benign masses; thus, no significant differences were found between the two tissue types. The difference might be because of the two reasons;

Firstly, it might be because of the model used by each study. [2, 3] demonstrated that as inflammation progresses within a pathological tissue, the complex structure of the tissue changes, resulting in increasing fluidity. These changes have been shown in the KVFD, Maxwell-FD, and microchannel flow models as power law behaviors exhibited by soft tissues[27, 30, 70, 71]. In contrast, the Voigt model does not show this characteristic.

Secondly, Visit Kumar et al. employed the SDUV technique, which is greatly affected by rapid attenuation of the amplitude of propagating shear waves due to media and dispersion geometry. Moreover, SDUV is limited to the frequency range (50Hz-400Hz) [13, 49, 60]. Above 400HZ, SDUV could not capture the shear wave dispersion due to attenuation. In the context of breast masses, benign and malignant tumors display considerable attenuation, limiting the observation of shear waves [60]. The characteristics of the surrounding tissue significantly affect geometric dispersion because the size difference between scatterers within the breast tissue and the wavelength of the propagating shear wave leads to the spreading of the shear wave, inducing challenges in displacement tracking [65]. Also, in small-sized masses, the shear wave travels inside the suspicious mass for a limited time. Thus, it challenges the estimation of viscoelastic parameters because it captures a few temporal points.

Additionally, USWE and its related techniques, like USDV, are limited in reaching the low-frequency range due to the tradeoff between resolution and Frequency of vibration of the shear wave [16, 65]. The resolution of the resulting map decreases as the vibration frequency decreases. Higher Frequency overcomes this challenge, but USDV used higher Frequency to acquire high resolutions and sacrificed penetration depth.

## 5. Limitation

However, there are several limitations to this review. Studies that utilized MRE in conjunction with the rheological model were not included due to a few studies that characterized the inflamed tissue’s stiffness. Secondly, the author did not perform a group comparison analysis of different data acquisition techniques because of the few studies obtained from three databases. For this reason, meta-analyses should be included in future review studies.

## 6. Future directions

Despite the emphasis made from the review regarding Viscosity and elasticity as the potential parameters for tissue characterization, further work should be considered to improve their performance. Although some models demonstrated remarkable performance in characterizing tissue biomechanical properties, the studies are still limited to reaching a concise agreement that specific models are considered the best. Researchers should work in VIVO to test their performance, or implementation of the more complex model should be considered. Moreover, researchers should consider improvements in hardware and software implementation and the reliability of signal acquisition methods and processing techniques of wave dispersion data because most USWE techniques face limitations with Frequency and penetration depth during data acquisition.

Researchers should also consider the measurement time constant (τ) of the KVFD, which has shown promising results in characterizing the stage of pathologies. Quantifying this parameter noninvasively and correlating it with available histopathological scoring systems used to stage steatosis and inflammation would be a novelty in clinical application.

## 7. Conclusion

The present systematic review focused on researching the association between elasticity and Viscosity in diseases, specifically as tissue inflammation progresses. Studies have shown that viscosity differs between normal tissue and tissue that has experienced long-term disease. However, no study has yet demonstrated that viscosity can vary between normal tissue and tissue in the early or initial stages of disease. The inconsistency of results from different methods regarding why the viscosity parameter still fails to differentiate between the stages of tissue from normal to early stages of disease and long-term diseased stages has been explained as a basis for enabling researchers to investigate further and improve the ability of this parameter to distinguish tissue stages. Conversely, assessing changes in elasticity and viscosity parameters as inflammation progresses will also be significant throughout treatment. Researchers may evaluate treatment responses and potentially optimize therapeutic strategies for better outcomes.

Moreover, it demonstrated the literature on utilizing mechanical rheological model, their application, and their clinical research limitations. Incorporating elasticity and viscosity measurements enhances the reliability and accuracy of diagnostic outcomes. However, achieving this requires using the appropriate model to extract tissue features. We have pointed out how existing models behave depending on the components of tissue microstructure. There is no consensus on mechanical rheological models within the clinical and elastography community. This review does not resolve which model is the best. Still, it has identified factors to consider when agreeing on the mechanical rheological model or implementing other complex models to characterize tissue inflammation. However, it has identified the mechanical rheological model that statistically performs best in quantifying viscoelastic parameters across various tissue pathologies.

## Supporting information

S1 File PRISMA 2020 abstract checklist. (DOCX)

S2 File PRISMA 2020 checklist. (DOCX)

S3 File ROB2 IRPG beta v9. (XLSM)
